# The Rejected Bill

**Published:** 1920-12-25

**Authors:** 


### THE REJECTED BILL.

In its minor shades, comment upon the unexpected action of the House of Lords in throwing out the Ministry of Health (Miscellaneous Provisions) Bill has been very varied. On the other hand, in its general trend, disappointment has been chiefly remarkable for its absence, and, in view of the very recent press boom in health matters generally, together with the noticeably increased interest of the public in its own hygienic welfare, it is, at first sight, a little surprising that this complacent attitude should be adopted towards so dramatic an ending.

Probably the fairest conclusion to be drawn is that, even without the influence of a volley of personal or political attacks upon bureaucracy and expenditure, the public was coming to see for itself how great were the individual problems dealt with by various sections of Dr. Addison's Bill, and how insistently some of them called for more representative consideration before they actually passed into law.

These sections we have discussed at various times. Never did they merit the sweeping condemnations vouchsafed them from extremist quarters, for, speaking generally, they were well intentioned and good in their essential points, if not wholly good. The Bill was a workmanlike measure, carrying in its admittedly unwieldy bulk a variety of concrete proposals for promoting the public health. But, as was to be expected almost from the beginning, although the theoretical and scientific grounds for support were numerous, those for political opposition and criticism from the specialist mind were as wide. The history of the Bill itself, its dropped and reclaimed clauses, and the local storms which it created, are too well known to call for further comment, but when a Bill with such a history is put before the Lords in the last few crowded days of the session, with or without press campaigns, it is not really surprising that temporarily it meets its end. We see it claimed that the Government itself must take the blame for the whole *débâcle*; in truth, it is remarkably difficult to lay it at any other door.

As to the future, unbiased by any political interest whatever, we can only hope that Dr. Addison will not thus again court disaster to the practical realisation of a number of urgent health reforms. To bring back a slightly modified measure, but still in its so-called omnibus form, seems simply to be offering the other cheek. The one genuine criticism running over the past many weeks has been that more true and more really expert specialised opinion should be deliberately brought forward and heard before final decisions are attempted. Whatever view be taken of the Bill's rejection, the event at least provides opportunity for this more ample and representative type of opinion to come to light, and we consider that these matters should be dealt with in the form of two or even three separate Bills; one, for example, dealing with housing and allied questions; one with local authorities and the "Poor-law" hospital as its main feature; and one to include, but not obscure, the provisions concerning amendment of the lunacy laws.

So brief an outline is merely indicative of a principle, but it is one, the necessity for which, the clauses as they affected the medical profession and the hospitals brought into more than noticeable prominence. In this connection, we have the satisfaction that the Minister has repeatedly called upon or received donations from professional men concerned, and, at the present time, a fresh mechanism for collecting representative opinion is being set up, but will the Government, even now, actually hear collective medical opinion? We have noted medical conferences galore, organised first by this body then by that. Occasionally some element of agreement has certainly been noticeable, when the matter concerned the meeting of a common "foe," but in the pure discussion of national health reforms for this country, power to co-operate is not nowadays a strong point on the side of the medical organisations.

It may be justifiably pondered as to which body is really representative of medical opinion in this country? We do not attempt to answer the question; we have no particular brief to hold for the excellence of any one of them. The question of national hygiene is of far greater importance than that of internal medical politics, and in this interval which the Bill's rejection provides, there is no doubt that the medical profession, to do its best for the country, must put its own house in order. The technical skill is there, the knowledge, but true organisation is lacking. Hardly is it a team without a leader, but rather one which has lacked the organisation through which it can, as a genuine whole, be led.

In commenting recently upon the possibilities lying behind the scheme formulated in the Scottish Report, we urged the paramount necessity that the medical organisations should come together for the common good of both medicine itself and the nation. In connection with these remarks we would call attention to the letter on page 299 of our present issue. By its constitution and professed aims the Federation of Medical and Allied Societies appears to be admirably adapted as a channel through which our ideal should materialise.

